# Prospective comparison of [^68^Ga]Ga-FAPI-04 PET/MRI, [^18^F]FDG PET/CT, and contrast-enhanced MRI for predicting pathologic complete response after neoadjuvant chemotherapy in breast cancer

**DOI:** 10.1007/s00259-026-08091-y

**Published:** 2026-07-23

**Authors:** Yuming Luo, Xiao Zhang, Runze Li, Yalan Zeng, Yu Zhao, Lei Li, Bei Qian, Yunxiao Xiao, Mengting Li, Yaqi Zhao, Silin Xu, Qin Yang, Huanwei Zhang, Hengyu Chen, Chong Lu, Xiaoli Lan, Chunping Liu

**Affiliations:** 1 Department of Breast and Thyroid Surgery, Union Hospital, Tongji Medical College, Huazhong University of Science and Technology, 1277 Jiefang Avenue, Wuhan, 430022 Hubei China; 2 Department of Nuclear Medicine, Union Hospital, Tongji Medical College, Huazhong University of Science and Technology, 1277 Jiefang Avenue, Wuhan, 430022 Hubei China; 3 Hubei Key Laboratory of Molecular Imaging, Wuhan, China; 4 Key Laboratory of Biological Targeted Therapy, Ministry of Education, Wuhan, China; 5 Department of Breast and Thyroid Surgery, The Second Affiliated Hospital of Hainan Medical University, Haikou, China; 6 Department of Breast and Thyroid Surgery, Chongqing Hospital, Union Hospital, Tongji Medical College, Huazhong University of Science and Technology, Chongqing, China

**Keywords:** Breast cancer, Neoadjuvant chemotherapy, Pathologic complete response, [^68^Ga]Ga-FAPI-04, PET/MRI

## Abstract

**Purpose:**

Accurate assessment of pathologic complete response (pCR) after neoadjuvant chemotherapy (NAC) remains challenging in breast cancer. This prospective single-center study compared [^68^Ga]Ga-FAPI-04 PET/MRI, [^18^F]FDG PET/CT, and contrast-enhanced MRI for predicting pathologic response after NAC.

**Methods:**

Women with biopsy-confirmed stage II–III breast cancer underwent baseline and post-therapy [^68^Ga]Ga-FAPI-04 PET/MRI, [^18^F]FDG PET/CT, and contrast-enhanced MRI before surgery. PET parameters were analyzed for primary tumors and axillary nodes. Selected variables were evaluated using LASSO and receiver-operating-characteristic analyses. Stromal FAP expression was assessed immunohistochemically in paired specimens.

**Results:**

Twenty-four patients completed the protocol, yielding 25 primary lesions and 44 metastatic lymph nodes across 27 axillary compartments. The pCR rate was 60.00% for primary lesions and 72.73% for axillary nodes. For primary-lesion response, post-therapy [^68^Ga]Ga-FAPI-04 SUVmax showed the highest performance (AUC, 0.84; sensitivity, specificity, and accuracy, 80.00% each); the best [^18^F]FDG parameter was ΔTBR% (AUC, 0.747). For nodal response, post-therapy [^68^Ga]Ga-FAPI-04 SULmean showed the highest performance (AUC, 0.89; sensitivity, 91.67%; specificity, 81.25%; accuracy, 84.09%) and exceeded the best [^18^F]FDG parameter on DeLong testing. MRI AUCs were 0.733 and 0.770 for primary and nodal disease, respectively. Stromal FAP expression correlated positively with [^68^Ga]Ga-FAPI-04 SUVmax.

**Conclusion:**

Post-therapy [^68^Ga]Ga-FAPI-04 PET may provide useful adjunctive information for response assessment after NAC in breast cancer, particularly for axillary nodal evaluation, potentially supporting more individualized preoperative planning.

**Trial registration:**

ClinicalTrials.gov, NCT07553741, retrospectively registered.

**Supplementary Information:**

The online version contains supplementary material available at https://doi.org/10.1007/s00259-026-08091-y.

## Introduction

Neoadjuvant chemotherapy (NAC) has become a standard treatment approach for patients with biologically aggressive or locally advanced breast cancer, as it enables tumor downstaging, increases the likelihood of breast-conserving surgery, and allows in vivo assessment of treatment sensitivity [1]. Notably, achieving pathologic complete response (pCR) after NAC is associated with improved long-term outcomes, especially in HER2-positive and triple-negative subtypes, underscoring the clinical importance of accurate response assessment for both prognostication and subsequent therapeutic decision-making [2]. Recent NeoSTEEP recommendations have reinforced standardized definitions of pCR in neoadjuvant breast cancer trials, emphasizing consistent reporting of residual disease in both the breast and regional lymph nodes [3].

Breast MRI is the established imaging modality for monitoring response after NAC because of its soft-tissue contrast and sensitivity for residual enhancement [4]. However, accuracy varies by subtype, treatment regimen, and imaging pattern, and false-positive findings may occur because of fibrosis, inflammation, or residual ductal carcinoma in situ [5].

[^18^F]FDG PET provides complementary biological information by measuring glucose metabolism and has demonstrated utility in assessing treatment response in breast cancer, including the prediction of pCR [6]. FDG-based imaging also contributes to response-adapted therapeutic strategies, particularly in HER2-positive disease [7].

Fibroblast activation protein (FAP) is highly expressed in cancer-associated fibroblasts within the tumor stroma and plays a critical role in extracellular matrix remodeling, tumor invasion, immune modulation, and treatment resistance [8]. In breast cancer, accumulating evidence indicates that FAP-positive stromal cells are associated with biologically aggressive tumor phenotypes and poor clinical outcomes [9]. These biological characteristics provide a strong rationale for FAP-targeted molecular imaging. Compared with [^18^F]FDG, radiolabeled fibroblast activation protein inhibitors (FAPIs) typically demonstrate superior tumor-to-background contrast and may more accurately reflect stromal changes following therapy [10].

Prior prospective studies have evaluated FAP-targeted PET/CT or PET/MRI in breast cancer and NAC response assessment, demonstrating promising lesion detectability, high tumor-to-background contrast, and associations between FAPI uptake and pathologic response or stromal FAP expression [10–13]. More recent studies have further evaluated serial [^68^Ga]Ga-FAPI-04 PET/CT during NAC, molecular subtype-specific patterns of FAPI-based response prediction, and the comparative performance of [^68^Ga]Ga-FAPI-04 PET/CT versus [^18^F]FDG PET/CT for residual disease assessment after neoadjuvant therapy [14–16]. Nevertheless, prospective data directly comparing [^68^Ga]Ga-FAPI-04 PET/MRI with both [^18^F]FDG PET/CT and contrast-enhanced MRI for predicting pCR in the same clinical setting remain limited, particularly when primary breast lesions and axillary lymph nodes are assessed separately. The incremental value of the present study is therefore its prospective multimodality head-to-head design, separate breast and nodal response analyses, and histopathologic validation of FAP expression.

Therefore, this prospective study compared the diagnostic performance of [^68^Ga]Ga-FAPI-04 PET/MRI, [^18^F]FDG PET/CT, and contrast-enhanced MRI for predicting pathologic response after NAC in primary breast lesions and axillary lymph nodes and examined the histopathologic correlate of FAPI uptake.

## Material and methods

### Patients

This prospective, single-center diagnostic accuracy study was approved by the Ethics Committee of Union Hospital, Tongji Medical College, Huazhong University of Science and Technology, and registered at ClinicalTrials.gov (NCT07553741). The study was registered after participant enrollment had begun and is therefore retrospectively registered. From April to December 2025, 27 consecutive women with biopsy-confirmed breast cancer scheduled for NAC were enrolled after providing written informed consent.

Eligible patients were 18–70 y old, had pathologically confirmed primary breast cancer with stage T2-4N0M0 or T0-4N1-3M0 disease, had received no prior antitumor therapy, had ECOG performance status 0–1, and had adequate organ function. Key exclusion criteria were distant metastasis, contraindications to chemotherapy, surgery, or [^68^Ga]Ga-FAPI-04 PET/MRI, prior malignancy or antitumor treatment, active infection, autoimmune disease, serious psychiatric or cognitive impairment, and pregnancy or lactation.

### Sample size estimation

For this exploratory diagnostic accuracy study, sample size was estimated for ROC-based discrimination of pCR after NAC. Assuming an AUC of 0.80–0.85, a 2-sided α of 0.05, and 80% power, 20–24 evaluable patients were required; therefore, target enrollment was set at 27 to allow for dropout or incomplete imaging or pathology.

### Procedures, NAC protocols, and surgical methods

All patients underwent baseline [^68^Ga]Ga-FAPI-04 PET/MRI, [^18^F]FDG PET/CT, and contrast-enhanced MRI before NAC. Molecular subtype was determined from core biopsy specimens, and subtype-guided NAC was administered according to institutional practice and China Anti-Cancer Association guidelines: TCbHP for HER2-positive disease, EC-T plus anti-PD-1 immunotherapy for triple-negative disease, and EC-T for luminal/HER2-negative disease [17].

**Fig. 1:**
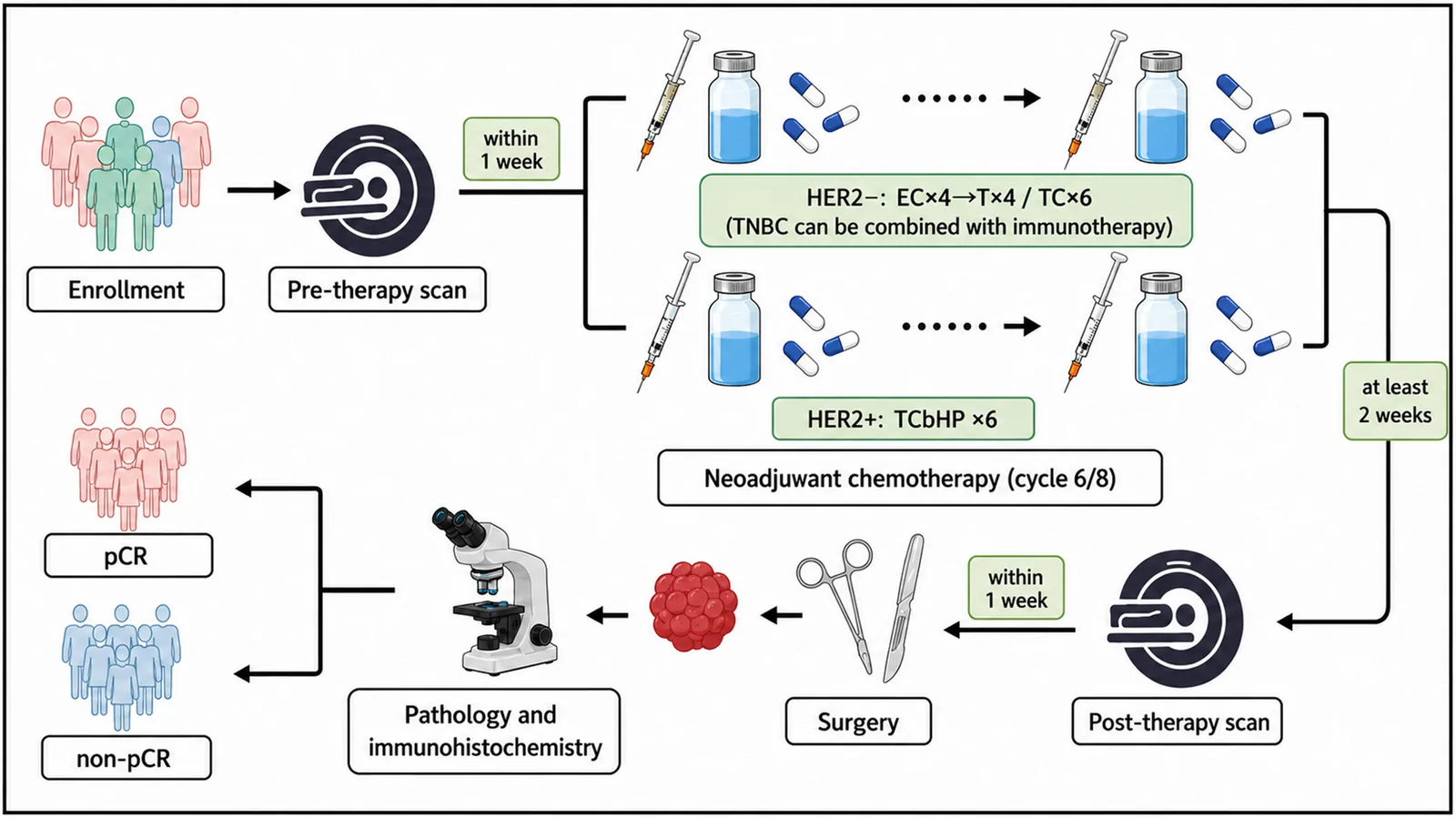
Procedures of the clinical trial

Two to three weeks after the final NAC cycle, all 3 imaging examinations were repeated, followed by surgery within 1 wk (Fig. 1). Surgical specimens were serially evaluated histopathologically. FAP immunohistochemistry was performed using a mouse anti-FAP antibody (Abmart, M079003; dilution, 1:900), and stromal FAP-positive area was scored as 0 (< 1%), 1 (1%−10%), 2 (11%−50%), or 3 (> 50%) [18, 19]. Axillary nodes were classified by levels I-III according to their relationship to the pectoralis minor muscle [20]. pCR was defined as absence of residual invasive carcinoma in the breast and axillary lymph nodes, irrespective of residual in situ disease (ypT0/isN0M0) [3].

### [^68^Ga]Ga-FAPI-04 PET/MRI, [^18^F]FDG PET/CT, and contrast-enhanced MRI acquisition

[^68^Ga]Ga-FAPI-04 PET/MRI was performed approximately 30 min after injection of 1.8–2.2 MBq/kg. Patients first underwent whole-body PET/MRI from the skull base to the upper thighs, followed by dedicated prone breast and axillary PET/MRI with both breasts suspended in a dedicated breast coil and an acquisition time of 8 min per bed position. [^18^F]FDG PET/CT was performed 60 min after injection of 3.7–5.5 MBq/kg using a Discovery VCT scanner (GE Healthcare). Contrast-enhanced MRI was performed on a 3.0-T Philips Ingenia system with T1-weighted, T2-weighted, and diffusion-weighted sequences. Imaging examinations were separated by at least 24 h. No adverse events related to imaging procedures occurred.

### Image interpretation

PET images were independently reviewed by 2 nuclear medicine physicians using PET VCAR software (GE Healthcare), with discrepancies resolved by a third physician. Volumes of interest were automatically delineated and adjusted as needed. Parameters included SUVmax, SUVmean, SULmax, SULmean, SULpeak, MTV, FTV, TLG, TLF, TBR, and percentage changes between baseline and post-therapy scans. TLG and TLF were calculated as SUVmean x MTV and SUVmean x FTV, respectively; TBR was lesion SUVmean divided by contralateral normal breast SUVmean. Lesion-level analysis included 25 primary lesions because 1 patient had occult breast cancer and 2 patients had 2 distinct lesions.

All imaging assessments were performed in a blinded manner. PET readers were blinded to histopathology, clinical outcomes, and other imaging findings; MRI was interpreted independently of PET and pathology; and pathologic evaluation was performed without knowledge of imaging findings.

MRI response assessment was based on tumor size, apparent diffusion coefficient, and residual enhancement in the tumor bed [4]. Nodal response was evaluated using morphologic and enhancement features, including cortical thickness, perinodal infiltration, fatty hilum status, and interval size change [21]. For nodal visual analysis, the unit was the axillary compartment; only baseline tracer-avid compartments were reassessed and were considered positive if at least 1 lymph node remained tracer-avid.

### Statistical analysis

Statistical analyses used R 4.5.2 and SPSS 26.0. Continuous variables were compared with the Student t test, Welch t test, or Mann–Whitney U test, and categorical variables with the χ2 test or Fisher-Freeman-Halton exact test. Significant univariable variables were explored by least absolute shrinkage and selection operator regression. ROC analysis calculated AUC, 95% CI, sensitivity, specificity, accuracy, and Youden index cutoffs; AUCs were compared by DeLong testing. False discovery rate was controlled using the Benjamini–Hochberg procedure for imaging-parameter comparison families. Two-sided *P* < 0.05 was considered significant. No indeterminate index-test or reference-standard results occurred. Patients with incomplete imaging, surgery, or pathology were excluded from the corresponding analyses.

## Results

### Patient characteristics

**Fig. 2:**
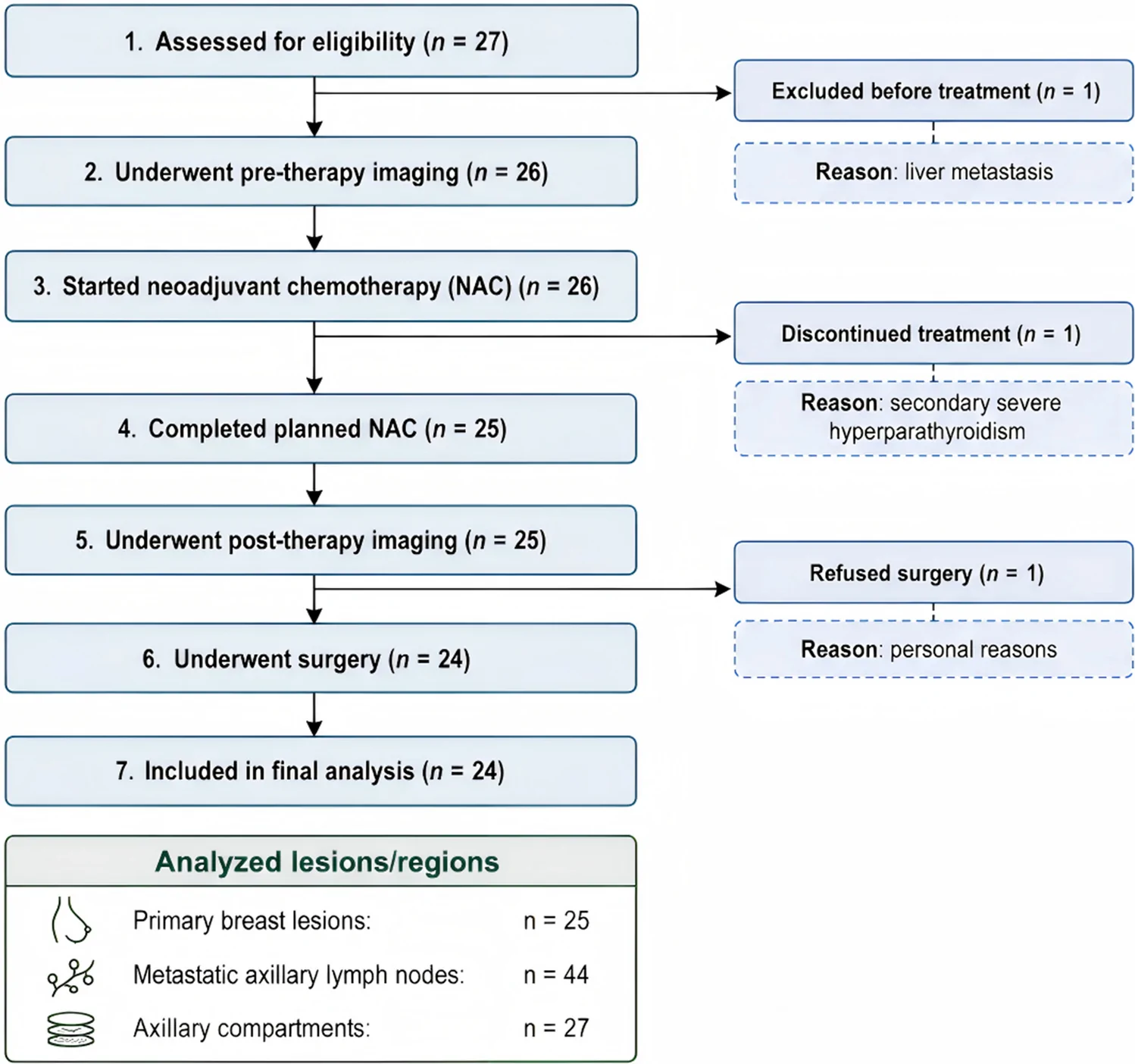
Trial profile

Of the 27 enrolled patients, 24 completed the protocol after one exclusion before treatment and two protocol non-completions (Fig. 2). Baseline characteristics are summarized in Table 1. All patients had AJCC stage II or III invasive ductal carcinoma; 1 had occult breast cancer and 5 had no baseline axillary nodal metastasis. Lesion-level analyses included 25 primary breast lesions, and imaging identified 44 metastatic lymph nodes across 27 axillary compartments before NAC.

**Table 1:** Patient characteristics (*n* = 24)

Characteristic	No. of patients
Mean age ± SD (y)	49.29 ± 9.35(range, 36–64)
Mean BMI ± SD	23.96 ± 2.08(range, 19.33–27.75)
Menstrual state	
Postmenopause	9(37.50%)
Premenopause	15(62.50%)
Clinical T category	
cT0	1(4.17%)
cT1	2(8.33%)
cT2	17(70.83%)
cT3	4(16.67%)
Clinical N category	
cN0	5(20.83%)
cN1	13(54.17%)
cN2	2(8.33%)
cN3	4(16.67%)
AJCC clinical stage	
Stage II (cT_0–1_N_1_M_0_/cT_2_N_0–1_M_0_/cT_3_N_0_M_0_)	17(70.83%)
Stage III (cT_3_N_1_M_0_/cT_4_N_0–1_M_0_/cT_0–4_N_2–3_M_0_)	7(29.17%)
Ki-67	
High expression	13(54.17%)
Low expression	11(45.83%)
Histological grading	
G2	7(29.17%)
G3	17(70.83%)
Molecular subtype	
Luminal	4(16.67%)
HER2 positive	12(50.00%)
Triple-negative	8(33.33%)
Pathologic response (all lesions, *n* = 24)	
pCR	13(54.17%)
Non-pCR	11(45.83%)
Pathologic response (breast primary lesions, *n* = 25)	
pCR	15(60.00%)
Non-pCR	10(40.00%)
Pathologic response (axillary lymph nodes, *n* = 44)	
pCR	32(72.73%)
Non-pCR	12(27.27%)

*HER2* Human Epidermal Growth Factor Receptor 2; *pCR* pathologic complete response. At lesion level, 25 primary breast lesions were analyzed because 1 patient had occult breast cancer without an identifiable breast lesion and 2 patients had 2 distinct primary lesions

### Pathology assessment

Among the 24 patients, 16 underwent modified radical mastectomy and 8 underwent breast-conserving surgery; 19 underwent axillary lymph node dissection and 5 underwent sentinel lymph node biopsy alone. Patient-level pCR was achieved in 13 of 24 patients (54.17%). Primary-lesion pCR occurred in 15 of 25 lesions (60.00%), and nodal pCR occurred in 32 of 44 metastatic lymph nodes (72.73%).

### Relationship between clinicopathologic characteristics and pCR

Associations between clinicopathologic variables and pathologic response after NAC are summarized in Online Resource 1, Table S1. No clinicopathologic factor was significantly associated with pCR in primary tumors. For axillary nodal response, the post-therapy neutrophil-to-lymphocyte ratio (NLR) was significantly lower in the pCR group compared to the non-pCR group (median: 2.17 vs. 2.72; *P* = 0.03). No significant association was observed for overall lesion pCR (Online Resource 1, Table S2).

### Visual assessment of post-therapy [^68^Ga]Ga-FAPI-04 and [^18^F]FDG PET

**Fig. 3:**
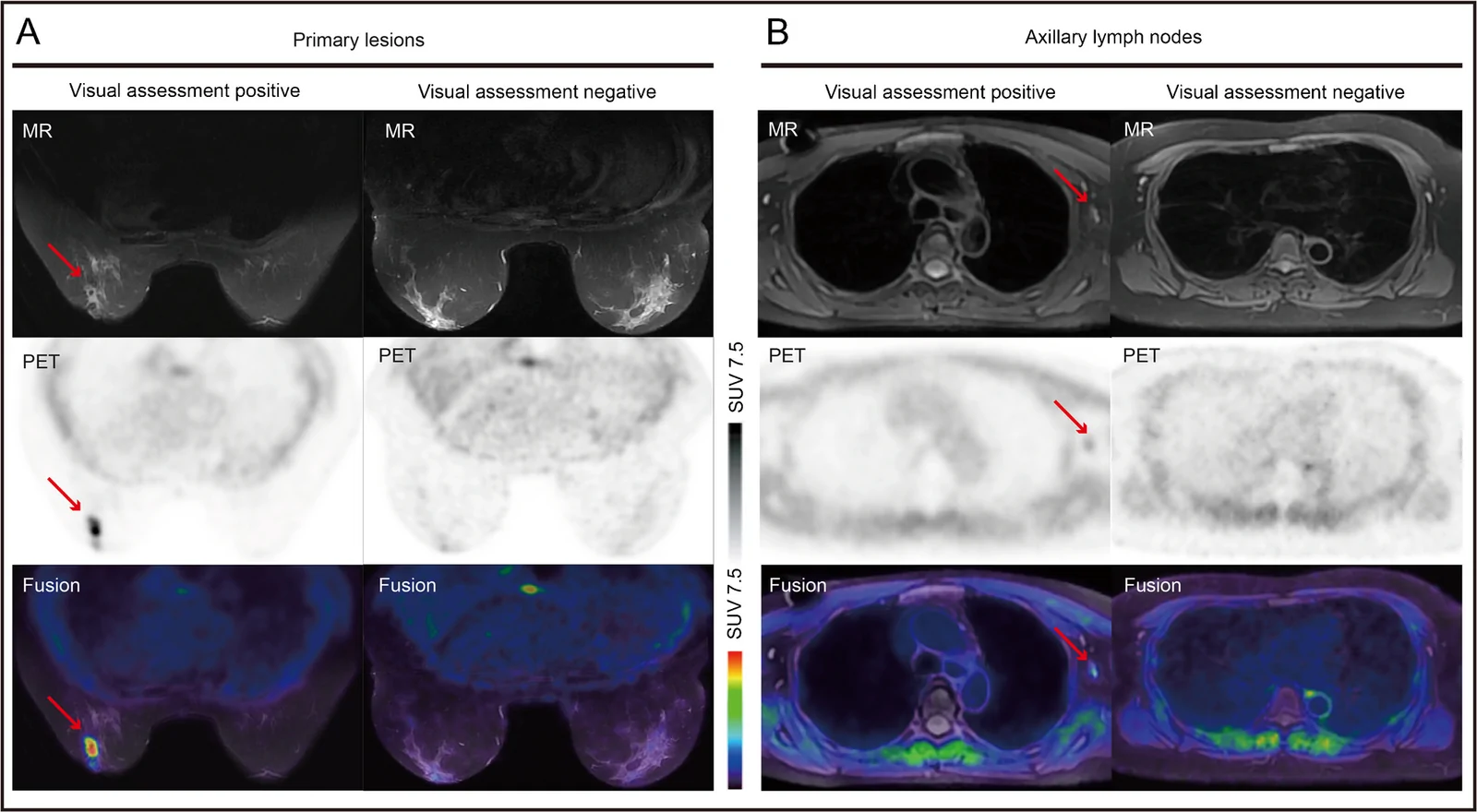
Post-therapy [^68^Ga]Ga-FAPI-04 PET/MRI images. **A** Visual assessment positive and negative for the primary lesion. **B** Visual assessment positive and negative for axillary lymph nodes

Post-therapy PET images were visually assessed for prediction of pathologic response. As shown in Table 2, for primary lesions, visual assessment using [^68^Ga]Ga-FAPI-04 showed higher accuracy than [^18^F]FDG, although the difference was not statistically significant. However, for compartment-level nodal pCR, [^68^Ga]Ga-FAPI-04 showed significantly higher diagnostic performance than [^18^F]FDG and was significantly associated with nodal pCR (*P* < 0.01), with a sensitivity of 87.50%, specificity of 84.21%, and accuracy of 85.19%. Representative cases are shown in Fig. 3.

**Table 2:** Visual assessment of post-therapy [^68^Ga]Ga-FAPI-04 and [^18^F]FDG PET

Lesions	Imaging parameter	Assessment results	Non-pCR	pCR	P	Sensitivity (%)	Specificity (%)	Accuracy (%)
Primary lesions	[^68^Ga]Ga-FAPI-04	Negative	0(0.00%)	6(100.00%)	0.05	100.00%	40.00%	64.00%
		Positive	10(52.63%)	9(47.37%)				
	[^18^F]FDG	Negative	1(20.00%)	4(80.00%)	0.62	90.00%	26.67%	52.00%
		Positive	9(45.00%)	11(55.00%)				
Compartment-level nodal	[^68^Ga]Ga-FAPI-04	Negative	1(5.89%)	16(94.12%)	< **0.01**	87.50%	84.21%	85.19%
		Positive	7(70.00%)	3(30.00%)				
	[^18^F]FDG	Negative	2(15.38%)	11(84.62%)	0.21	75.00%	57.89%	62.96%
		Positive	6(42.86%)	8(57.14%)

### Performance of [^68^Ga]Ga-FAPI-04 PET/MRI, [^18^F]FDG PET/CT, and contrast-enhanced MRI

In quantitative analyses (Online Resource 1, Table S3), no baseline [^68^Ga]Ga-FAPI-04 or [^18^F]FDG PET parameter was significantly associated with primary-lesion pCR. On post-therapy [^68^Ga]Ga-FAPI-04 imaging, SUVmax, SUVmean, SULmax, SULmean, SULpeak, and TBR were significantly lower in the pCR group (all *P* < 0.05), whereas no post-therapy [^18^F]FDG PET parameter showed a significant association. SUVmax, SULmax, SULmean and TBR also remained significant after false discovery rate correction, whereas SUVmean and SULpeak did not. For percentage changes, ΔFTV% and ΔTLF% on [^68^Ga]Ga-FAPI-04 PET, as well as ΔSULmax%, ΔSULpeak%, and ΔTBR% on [^18^F]FDG PET, were significantly higher in the pCR group before correction; however, after false discovery rate correction, only ΔSULpeak% and ΔTBR% remained statistically significant. (Online Resource 1, Table S4).

For axillary lymph nodes, baseline [^68^Ga]Ga-FAPI-04 TBR showed a nominal association with nodal pCR (7.84 ± 5.23 vs. 12.13 ± 5.23; *P* = 0.03) but did not remain significant after false discovery rate correction. On post-therapy imaging, all evaluated [^68^Ga]Ga-FAPI-04 parameters remained significantly lower in the pCR group, and the post-therapy [^68^Ga]Ga-FAPI-04 parameters remained significant after false discovery rate correction (all q ≤ 0.038). Among percentage-change metrics, ΔSUVmean%, ΔSULmean%, ΔFTV%, and ΔTLF% also remained significant after false discovery rate correction, whereas ΔSUVmax%, ΔSULmax%, and ΔSULpeak% did not (Online Resource 1, Table S4).

Contrast-enhanced MRI detected all primary lesions and all axillary metastases at baseline. After treatment, 13 of 25 primary lesions were MRI-negative and 12 were MRI-positive; pCR was observed in 11 MRI-negative lesions, whereas 8 MRI-positive lesions exhibited residual disease (*P* < 0.05). Among 27 axillary compartments, 17 were MRI-negative and 10 were MRI-positive; nodal pCR was observed in 15 MRI-negative compartments, whereas residual metastatic lymph nodes were identified in 6 MRI-positive compartments (*P* < 0.05).

As shown in Online Resource 1, Table S5, post-therapy [^68^Ga]Ga-FAPI-04 TLR showed a nominal association with overall-lesion pCR (*P* < 0.05), whereas no [^18^F]FDG parameter was associated with overall-lesion pCR. This association did not remain significant after false discovery rate correction (Online Resource 1, Table S4).

### Receiver-operating-characteristic analyses of imaging and clinical parameters for pCR

Variables were entered into LASSO regression for feature selection (Online Resource 2, Figs. S1 and S2). For [^68^Ga]Ga-FAPI-04, post-therapy SUVmax, SULmean, and TBR were strongly associated with primary-lesion pCR, whereas post-therapy SULmean and ΔTLF% were associated with nodal pCR. For [^18^F]FDG, only ΔTBR% was associated with primary-lesion pCR; no significant parameter was identified for nodal pCR, and therefore LASSO analysis was not performed for nodal [^18^F]FDG parameters.

**Fig. 4:**
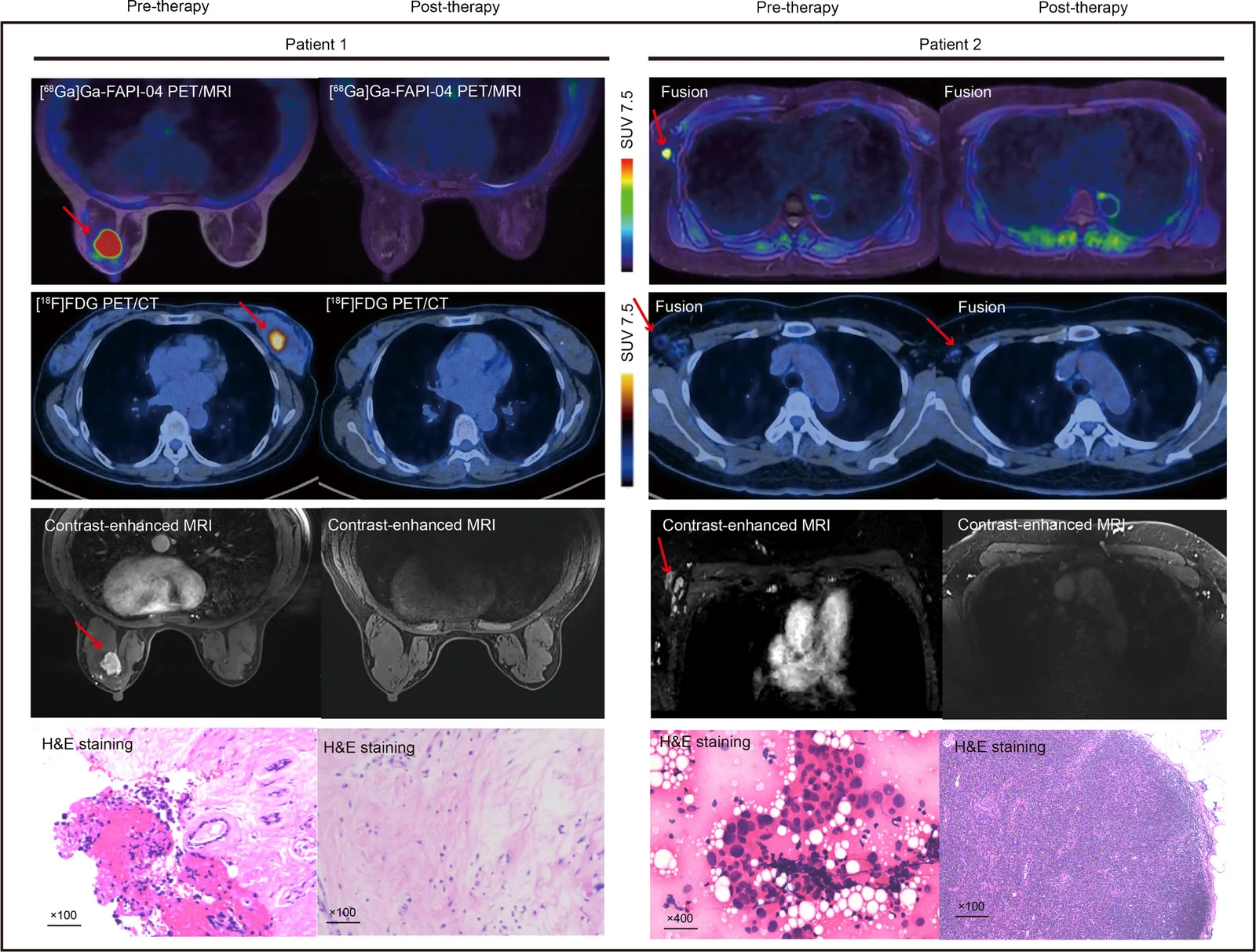
Representative cases of primary and axillary nodal pCR after neoadjuvant chemotherapy. Patient 1 A 54-y-old woman with invasive breast cancer who achieved primary pCR after NAC. Pre-therapy [^68^Ga]Ga-FAPI-04 PET/MRI, [^18^F]FDG PET/CT, and contrast-enhanced MRI show the primary lesion (red arrows). Post-therapy images show no residual uptake or enhancement, and surgical pathology confirmed primary pCR. Patient 2 A 50-y-old woman with biopsy-proven axillary nodal metastasis who achieved axillary pCR after NAC. Pre-therapy images show metastatic axillary nodes (red arrows). Post-therapy [^68^Ga]Ga-FAPI-04 PET/MRI and contrast-enhanced MRI show no residual nodal abnormality, whereas [^18^F]FDG PET/CT shows residual nodal uptake with ΔSULmax% below the cutoff, yielding a false-positive non-pCR prediction. Surgical pathology confirmed nodal pCR

ROC analysis identified the optimal parameters for predicting primary-lesion pCR (Online Resource 2, Fig. S3a, Table 3). Post-therapy [^68^Ga]Ga-FAPI-04 SUVmax showed the highest diagnostic performance (AUC, 0.84; 95% CI, 0.683–0.997; sensitivity, 80.00%; specificity, 80.00%; accuracy, 80.00%), whereas ΔTBR% was the best-performing [^18^F]FDG parameter (AUC, 0.747; 95% CI, 0.545–0.949; sensitivity, 70.00%; specificity, 80.00%; accuracy, 76.00%). DeLong testing showed no significant difference between the 2 tracers (*P* > 0.05). For prediction of axillary nodal pCR (Online Resource 2, Fig. S3b, Table 3), post-therapy [^68^Ga]Ga-FAPI-04 SULmean showed the highest diagnostic performance (AUC, 0.89; 95% CI, 0.7765–1.000; sensitivity, 91.67%; specificity, 81.25%; accuracy, 84.09%), whereas ΔSULmax% was the best-performing [^18^F]FDG-derived parameter (AUC, 0.669; 95% CI, 0.4875–0.851; sensitivity, 83.33%; specificity, 53.13%; accuracy, 61.36%). DeLong testing showed that [^68^Ga]Ga-FAPI-04 had significantly higher discriminative performance than [^18^F]FDG for nodal pCR (*P* < 0.05). Representative cases are shown in Fig. 4.

**Table 3:** Diagnostic performance for prediction of pCR

Lesions	Parameter	Subparameter	Cutoff	AUC	P	95% CI	Sensitivity (%)	Specificity (%)	Accuracy (%)
Primary lesions	Contrast-enhanced MRI in post-therapy		—	0.733	**0.04**	0.528–0.938	80.00%	66.67%	72.00%
	[^68^Ga]Ga-FAPI-04	Post-therapy SUVmax	2.395	0.84	< **0.01**	0.683–0.997	80.00%	80.00%	80.00%
	[^18^F]FDG	ΔTBR%	59.05	0.747	**0.04**	0.545–0.949	70.00%	80.00%	76.00%
Axillary nodes	Contrast-enhanced MRI in post-therapy		—	0.77	**0.01**	0.563–0.977	75.00%	78.95%	77.78%
	[^68^Ga]Ga-FAPI-04	Post-therapy SULmean	0.36	0.89	< **0.01**	0.7765–1	91.67%	81.25%	84.09%
	[^18^F]FDG	ΔSULmax%	89.51	0.669	0.08	0.4875–0.851	83.33%	53.13%	61.36%
	NLR		2.35	0.82	**0.03**	0.6231–1	100.00%	61.54%	73.68%

*AUC* area under the curve; *CI* confidence interval; *NLR* neutrophil-to-lymphocyte ratio; *pCR* pathologic complete response; *TBR* tumour-to-background ratio

### Association between FAP immunohistochemistry and [^68^Ga]Ga-FAPI-04 uptake

**Fig. 5:**
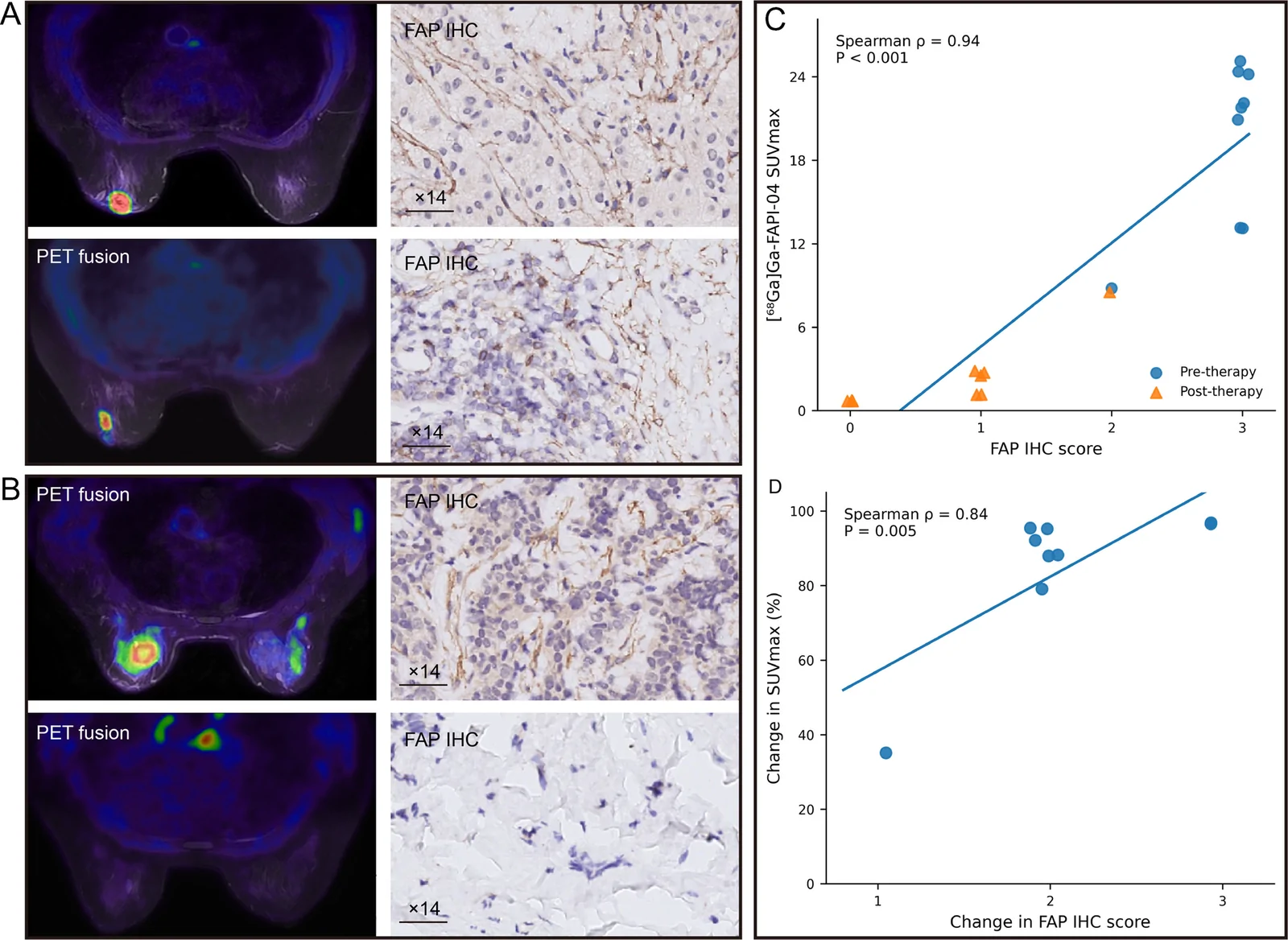
Relationship between [^68^Ga]Ga-FAPI-04 uptake and stromal FAP expression. **A** Patient 1 showed high baseline uptake and FAP expression (SUVmax, 21.79; IHC score, 3) and residual uptake with reduced FAP expression after chemotherapy without pCR (SUVmax, 8.53; IHC score, 1). **B** Patient 2 showed moderate baseline uptake and FAP expression (SUVmax, 8.80; IHC score, 1), with no visible post-therapy uptake and no residual FAP expression after pCR (IHC score, 0). **C** SUVmax correlated positively with FAP IHC score (Spearman *ρ* = 0.94, *P* < 0.001). **D** Percentage decrease in SUVmax correlated positively with decrease in FAP IHC score after chemotherapy (Spearman *ρ* = 0.84, *P* = 0.005). IHC = immunohistochemistry

Paired pre- and post-therapy specimens from 9 patients were evaluated for FAP expression. [^68^Ga]Ga-FAPI-04 SUVmax correlated positively with stromal FAP score, and reduction in SUVmax correlated with reduction in FAP expression after NAC. FAP expression was minimal in pCR tissue and decreased in residual tumors after chemotherapy, supporting a biologic basis for FAPI uptake changes (Fig. 5).

### Subgroup analysis for diagnostic performance

Patients were stratified according to HER2 status for exploratory, hypothesis-generating subgroup analyses of primary-lesion pCR (Online Resource 1, Tables S6 and S7; Online Resource 2, Fig. S4). In HER2-positive patients, baseline [^68^Ga]Ga-FAPI-04 SUVmean and post-therapy [^18^F]FDG MTV showed apparently high diagnostic performance, with no significant difference between the 2 tracers on DeLong analysis. In HER2-negative patients, no [^18^F]FDG parameter was significantly associated with pCR, whereas ΔSUVmax% on [^68^Ga]Ga-FAPI-04 showed a high AUC. Within subgroup-specific comparison families, the nominally significant HER2-positive and HER2-negative associations remained significant after false discovery rate correction; however, because the subgroup sample sizes were very small, these findings should be interpreted cautiously as exploratory and should not be considered definitive evidence of subtype-specific diagnostic performance (Online Resource 1, Table S4).

## Discussion

Accurate identification of pathologic complete response (pCR) after neoadjuvant chemotherapy (NAC) has direct clinical relevance in breast cancer because it informs prognosis and may influence surgical decision-making, particularly in the axilla [2, 3]. In this prospective head-to-head study, post-therapy [^68^Ga]Ga-FAPI-04 PET showed the strongest association with pathologic response among the three imaging modalities evaluated. Specifically, post-therapy SUVmax was the optimal parameter for assessing response in primary lesions, whereas post-therapy SULmean yielded the highest diagnostic performance for axillary lymph nodes. In comparison, the best [^18^F]FDG-derived metrics showed only moderate discriminative ability, and contrast-enhanced MRI, although clinically useful, provided less diagnostic information than the strongest [^68^Ga]Ga-FAPI-04-derived parameters. These findings suggest that FAP-targeted imaging may offer a biologically meaningful approach for response assessment after NAC, particularly when viable tumor burden is low but treatment-related stromal remodeling remains active [8, 9].

Our results are consistent with the growing body of evidence supporting FAPI-based imaging in breast cancer. Backhaus et al. reported promising initial results using [^68^Ga]Ga-FAPI-46 PET/MRI for response assessment after NAC, showing excellent correlation between follow-up FAPI uptake and pathologic response status [11]. Chen et al. further showed in a prospective study that [^68^Ga]-labeled FAPI PET/CT could predict early and late pathologic response to NAC in breast cancer [13]. Zheng et al. extended this evidence by showing that serial [^68^Ga]Ga-FAPI-04 PET/CT facilitated early prediction of pathological response and evaluation of therapeutic efficacy during NAC [14]. Chen et al. reported that the predictive value of [^68^Ga]Ga-FAPI-04 PET/CT differed across breast cancer molecular subtypes [15]. Zhu et al. showed in clinically node-positive breast cancer that preoperative [^68^Ga]Ga-FAPI-04 PET/CT had higher sensitivity than [^18^F]FDG PET/CT for identifying residual disease after neoadjuvant therapy, with comparable specificity [16]. Other clinical studies and reviews have highlighted the potential of FAPI imaging for improving lesion conspicuity, tumor-to-background contrast, and clinical management in breast cancer [10, 12, 22]. Building on these prior studies, the present work adds prospective head-to-head evidence by comparing [^68^Ga]Ga-FAPI-04 PET/MRI, [^18^F]FDG PET/CT, and contrast-enhanced MRI within the same response assessment after NAC framework and by evaluating primary breast lesions and axillary lymph nodes separately.

A key strength of the present study is the separate evaluation of primary-tumor and axillary nodal response. For primary lesions, the best [^68^Ga]Ga-FAPI-04-derived parameter numerically exceeded the best [^18^F]FDG-derived parameter, although the difference did not reach statistical significance. This pattern may reflect the biologic complexity of the tumor bed after NAC, where residual fibrosis, isolated tumor cells, and therapy-induced inflammatory changes can confound image interpretation across modalities. In contrast, for axillary lymph nodes, [^68^Ga]Ga-FAPI-04 imaging showed clearer qualitative and quantitative separation than [^18^F]FDG. This finding is clinically relevant because axillary status after NAC remains a critical determinant of prognosis and guides locoregional management, whereas nodal restaging with conventional imaging remains challenging after NAC [21, 23].

Clinically, improved assessment after NAC of residual disease may help refine preoperative planning for both the breast and axilla. For primary tumors, more accurate imaging may inform the feasibility of breast-conserving surgery, the extent of resection, and postoperative systemic treatment decisions. This is particularly relevant because breast surgery de-escalation after excellent response to NAC, including omission of surgery in highly selected exceptional responders, is being explored only when residual disease can be reliably excluded by rigorous post-therapy assessment [24]. Although [^68^Ga]Ga-FAPI-04 PET was not statistically superior to [^18^F]FDG PET for primary-lesion pCR in this cohort, its numerically higher performance suggests potential complementary value in the post-therapy tumor bed, where fibrosis, inflammation, and scattered residual disease may complicate conventional assessment. The stronger performance for axillary nodal response is also clinically important because residual nodal disease directly affects axillary surgery and subsequent locoregional treatment decisions, particularly in the context of ongoing axillary de-escalation efforts [25, 26]. Thus, [^68^Ga]Ga-FAPI-04 PET may contribute to integrated preoperative assessment of residual disease burden, but these findings remain complementary rather than practice-changing and cannot replace surgical pathologic evaluation.

The favorable performance of [^68^Ga]Ga-FAPI-04 imaging is biologically plausible. In paired tissue analyses, stromal FAP expression correlated with [^68^Ga]Ga-FAPI-04 SUVmax, and pCR tissues showed minimal residual FAP expression. These results support the concept that declining FAPI uptake reflects reduced stromal activation after effective therapy [9, 27].

MRI showed significant predictive utility for both breast and nodal pCR but can be limited by fibrosis, inflammation, and nonmass enhancement [4, 5, 28]. The [^18^F]FDG PET results also warrant cautious interpretation. Although no post-therapy [^18^F]FDG parameter significantly predicted pCR in the primary lesion on univariable analysis, dynamic [^18^F]FDG markers such as ΔTBR% retained moderate diagnostic performance, consistent with prior evidence supporting serial [^18^F]FDG-based metrics for pCR prediction [6, 29]. [^18^F]FDG PET continues to play an important role in response-adapted strategies, particularly in HER2-positive disease [7, 30]. Exploratory data from the PHERGain trial also highlight the complementary value of [^18^F]FDG PET relative to breast MRI for predicting pCR and stratifying outcomes in early-stage HER2-positive disease [31]. Nevertheless, compared with [^18^F]FDG, [^68^Ga]Ga-FAPI-04 PET may provide higher contrast for detecting residual stromal activity and may be less influenced by biologic and physiologic confounders related to glucose metabolism.

The HER2-stratified analyses were exploratory and hypothesis-generating only. In HER2-positive tumors, [^68^Ga]Ga-FAPI-04 and [^18^F]FDG showed comparable apparent discriminative ability for predicting primary-lesion pCR. In HER2-negative disease, no [^18^F]FDG parameter was significantly associated with pCR, whereas a [^68^Ga]Ga-FAPI-04-derived percentage-change parameter showed a high AUC. Because of the limited sample size, these subgroup findings should be regarded as hypothesis-generating rather than practice-changing. Even so, they are consistent with prior observations that the predictive accuracy of [^18^F]FDG PET varies across molecular subtypes and treatment contexts [29], and with recent evidence suggesting subtype-dependent predictive patterns of [^68^Ga]Ga-FAPI-04 PET/CT during NAC [15]. These data suggest that stromal-targeted imaging may be particularly useful in subgroups for which tumor-cell glucose metabolism is not a stable indicator of treatment response.

This study has limitations. First, the sample size was modest and enrollment was single-center, limiting statistical power and generalizability. Second, the trial was retrospectively registered, although the study was designed prospectively and the imaging protocol, reference standard, and main analyses were prespecified before completion of data analysis. Third, combined patient-, lesion-, and compartment-level analyses may introduce statistical dependence. Fourth, the primary-lesion sample was small and the pCR rate was relatively high, reducing precision. Fifth, subgroup analyses, including the HER2-stratified analyses, were exploratory and hypothesis-generating only. Sixth, MRI interpretation was based on routine clinical assessment rather than fully quantitative multiparametric or radiomics methods. Larger multicenter studies with validation cohorts are needed.

Overall, our data support post-therapy [^68^Ga]Ga-FAPI-04 PET as a promising imaging biomarker for assessing treatment response after NAC in breast cancer, particularly for evaluating axillary nodal clearance while also providing potentially useful complementary information for the primary tumor bed. Larger multicenter studies are needed to confirm the optimal imaging time point and quantitative thresholds and to determine whether integration of [^68^Ga]Ga-FAPI-04 PET with MRI, molecular subtyping, and circulating biomarkers can improve personalized surgical and systemic treatment decision-making.

In patients with breast cancer undergoing NAC, post-therapy [^68^Ga]Ga-FAPI-04 PET showed promising performance for predicting pathologic response. It showed numerically higher diagnostic performance than [^18^F]FDG PET for primary lesions and significantly higher discriminative performance for axillary lymph nodes. Concordance between FAPI uptake and stromal FAP expression supports the biologic validity of this approach. [^68^Ga]Ga-FAPI-04 PET may therefore serve as a valuable adjunct for response assessment after NAC, particularly in the axilla, pending confirmation in larger multicenter cohorts.

## Supplementary Information

Below is the link to the electronic supplementary material.

Supplementary file 1 (DOCX 81 KB)

Supplementary file 2 (DOCX 3519 KB)

## Data Availability

The datasets generated during and/or analysed during the current study are not publicly available due to patient privacy and institutional restrictions but are available from the corresponding author on reasonable request and subject to institutional and ethical approval.

## Declarations

### Clinical trial registration

ClinicalTrials.gov, NCT07553741, retrospectively registered.

## References

[1] Korde LA, Somerfield MR, Carey LA, et al. Neoadjuvant chemotherapy, endocrine therapy, and targeted therapy for breast cancer: ASCO guideline. J Clin Oncol. 2021;39:1485–505. 10.1200/JCO.20.03399. PMC8274745 33507815

[2] Spring LM, Fell G, Arfe A, et al. Pathologic complete response after neoadjuvant chemotherapy and impact on breast cancer recurrence and survival: a comprehensive meta-analysis. Clin Cancer Res. 2020;26:2838–48. 10.1158/1078-0432. PMC7299787 32046998

[3] Litton JK, Regan MM, Pusztai L, et al. Standardized definitions for efficacy end points in neoadjuvant breast cancer clinical trials: NeoSTEEP. J Clin Oncol. 2023;41:4433–42. 10.1200/JCO.23.00435. PMC10522109 37433103

[4] Reig B, Lewin AA, Du L, et al. Breast MRI for evaluation of response to neoadjuvant therapy. Radiographics. 2021;41:665–79. 10.1148/rg.2021200134. 33939542

[5] Hu Q, Hu Y, Ai H, Xia L, Liu R, Ai T. Preoperative MRI in evaluating pathologic complete response to neoadjuvant chemotherapy in patients with breast cancer: a study focused on influencing factors of baseline clinical-pathological and imaging features. Front Oncol. 2024;14:1366613. 10.3389/fonc.2024.1366613. PMC11140022 38826784

[6] Wu Y, Li Y, Chen B, et al. 18F-FDG PET/CT for early prediction of pathological complete response in breast cancer neoadjuvant therapy: a retrospective analysis. Oncologist. 2024;29:e1646–55. 10.1093/oncolo/oyae185. PMC11630790 39045652

[7] Pérez-García JM, Cortés J, Ruiz-Borrego M, et al. 3-year invasive disease-free survival with chemotherapy de-escalation using an ^18^F-FDG-PET-based, pathological complete response-adapted strategy in HER2-positive early breast cancer (PHERGain): a randomised, open-label, phase 2 trial. Lancet. 2024;403:1649–59. 10.1016/S0140-6736(24)00054-0. 38582092

[8] Lee HH, Al-Ogaili Z. Fibroblast activation protein and the tumour microenvironment: challenges and therapeutic opportunities. Oncol Rev. 2025;19:1617487. 10.3389/or.2025.1617487. PMC12308159 40741380

[9] Chen W, Jiang M, Zou X, et al. Fibroblast activation protein (FAP)^+^ cancer-associated fibroblasts induce macrophage M2-like polarization via the fibronectin 1-integrin α5β1 axis in breast cancer. Oncogene. 2025;44:2396–412. 10.1038/s41388-025-03359-3. 40263422

[10] Li T, Zhang J, Yan Y, Tan M, Chen Y. Applications of FAPI PET/CT in the diagnosis and treatment of breast and the most common gynecologic malignancies: a literature review. Front Oncol. 2024;14:1358070. 10.3389/fonc.2024.1358070. PMC10949888 38505595

[11] Backhaus P, Burg MC, Asmus I, et al. Initial results of [^68^Ga]Ga-FAPI-46 PET/MRI to assess response to neoadjuvant chemotherapy in breast cancer. J Nucl Med. 2023;64:717–23. 10.2967/jnumed.122.264871. PMC10152127 36396458

[12] Guo W, Xu W, Meng T, et al. FAP-targeted PET/CT imaging in patients with breast cancer from a prospective bi-center study: insights into diagnosis and clinic management. Eur J Nucl Med Mol Imaging. 2025;52:2317–34. 10.1007/s00259-025-07108-2. 39883140

[13] Chen L, Zheng S, Chen L, et al. ^68^Ga-labeled fibroblast activation protein inhibitor PET/CT for the early and late prediction of pathologic response to neoadjuvant chemotherapy in breast cancer patients: a prospective study. J Nucl Med. 2023;64(12):1899–905. 10.2967/jnumed.123.266079. PMC10690122 37918866

[14] Zheng S, Xu F, Chen Y, et al. The value of serial [^68^Ga]Ga-FAPI-04 PET/CT in predicting pathological response and evaluating therapeutic efficacy to neoadjuvant chemotherapy in breast cancer. Eur J Nucl Med Mol Imaging. 2025;53(1):362–73. 10.1007/s00259-025-07444-3. 40650678

[15] Chen L, Kong L, Yang J, et al. [^68^Ga]Ga-labeled fibroblast activation protein inhibitor PET/CT for monitoring neoadjuvant chemotherapy responses in breast cancer subtypes. Breast Cancer Res. 2026;28(1):82. 10.1186/s13058-026-02267-y. PMC13151235 41904503

[16] Zhu S, Jin Y, Hu C, et al. Diagnostic performance of ^68^Ga-FAPI PET/CT versus ^18^F-FDG PET/CT in evaluating neoadjuvant therapy pathological response in breast cancer. NPJ Breast Cancer. 2026. 10.1038/s41523-026-00983-4. PMC13534706 42230653

[17] Society of Breast Cancer, China Anti-Cancer Association; Breast Oncology Group of the Oncology Branch of the Chinese Medical Association. Guidelines for breast cancer diagnosis and treatment by China Anti-Cancer Association (2024 edition). China Oncol. 2023;33(12):1092–1187. 10.19401/j.cnki.1007-3639.2023.12.004.

[18] Kim HM, Jung WH, Koo JS. Expression of cancer-associated fibroblast related proteins in metastatic breast cancer: an immunohistochemical analysis. J Transl Med. 2015;13:222. 10.1186/s12967-015-0587-9. PMC4702422 26163388

[19] Xu W, Zou Y, Zhang Y, et al. The clinical value of FAPI PET/CT in delineating target volumes for nasopharyngeal carcinoma. Eur J Nucl Med Mol Imaging. 2025;53(1):105–15. 10.1007/s00259-025-07347-3. PMC12660409 40447840

[20] Chang JM, Leung JWT, Moy L, Ha SM, Moon WK. Axillary nodal evaluation in breast cancer: state of the art. Radiology. 2020;295:500–15. 10.1148/radiol.2020192534. 32315268

[21] Di Paola V, Mazzotta G, Pignatelli V, et al. Beyond N staging in breast cancer: importance of MRI and ultrasound-based imaging. Cancers (Basel). 2022;14:4270. 10.3390/cancers14174270. PMC9454572 36077805

[22] Sahin E, Kus T, Aytekin A, et al. ^68^Ga-FAPI PET/CT as an alternative to ^18^F-FDG PET/CT in the imaging of invasive lobular breast carcinoma. J Nucl Med. 2024;65:512–9. 10.2967/jnumed.123.266798. 38485276

[23] Di Micco R, Hartmann S, Banys-Paluchowski M, et al. Diagnostic performance of axillary ultrasound after neoadjuvant chemotherapy in initially node-positive breast cancer patients - results from the prospective AXSANA registry trial. Eur J Cancer. 2025;226:115607. 10.1016/j.ejca.2025.115607. 40645031

[24] Kuerer HM, Smith BD, Krishnamurthy S, et al. Eliminating breast surgery for invasive breast cancer in exceptional responders to neoadjuvant systemic therapy: a multicentre, single-arm, phase 2 trial. Lancet Oncol. 2022;23:1517–24. 10.1016/S1470-2045(22)00613-1. 36306810

[25] Park WK, Nam SJ, Kim SW, et al. Axillary surgery in breast cancer: evidence-based de-escalation across upfront and post-neoadjuvant settings. J Breast Cancer. 2026;29:17–32. 10.4048/jbc.2025.0290. PMC12961323 41612659

[26] Capasso K, Mitri S, Roldan-Vasquez E, et al. Axillary de-escalation after neoadjuvant chemotherapy for advanced lymph node involvement in breast cancer. Am J Surg. 2024;236:115893. 10.1016/j.amjsurg.2024.115893. 39153469

[27] Jia H, Chen X, Zhang L, Chen M. Cancer-associated fibroblasts in cancer development and therapy. J Hematol Oncol. 2025;18:36. 10.1186/s13045-025-01688-0. PMC11954198 40156055

[28] Romeo V, Accardo G, Perillo T, et al. Assessment and prediction of response to neoadjuvant chemotherapy in breast cancer: a comparison of imaging modalities and future perspectives. Cancers (Basel). 2021;13:3521. 10.3390/cancers13143521. PMC8303777 34298733

[29] Vorster M, Sathekge M. ^18^F-FDG PET/CT for prediction of response in breast cancer. Semin Nucl Med. 2026;56:86–96. 10.1053/j.semnuclmed.2025.11.019. 41513559

[30] Gebhart G, Keyaerts M, Guiot T, et al. Optimal [^18^F]FDG PET/CT cutoff for pathologic complete response in HER2-positive early breast cancer patients treated with neoadjuvant trastuzumab and pertuzumab in the PHERGain trial. J Nucl Med. 2024;65:708–13. 10.2967/jnumed.123.266384. 38575192

[31] García-Mosquera JJ, Pérez-García JM, Ruiz-Borrego M, et al. Comparing [^18^F]FDG-positron emission tomography and breast magnetic resonance imaging to predict pathological complete response and 3-year invasive disease-free survival in HER2-positive early breast cancer patients: an unplanned exploratory analysis of the PHERGain trial. ESMO Open. 2025;10:105875. 10.1016/j.esmoop.2025.105875. PMC12639436 41202503

