# Supplementary material for: Prospective comparison of [68Ga]Ga-FAPI-04 PET/MRI, [18F]FDG PET/CT, and contrast-enhanced MRI for predicting pathologic complete response after neoadjuvant chemotherapy in breast cancer
Source: Eur J Nucl Med Mol Imaging. 2026 Jul 23;53(12):7221–32. doi: 10.1007/s00259-026-08091-y (PMC13633318; doi:10.1007/s00259-026-08091-y)
Supplement: Supplementary file 1 [file 259_2026_8091_MOESM1_ESM.docx]

Online Resource 1 Table S1

| Characteristic | Patients with  non-pCR(primary lesions) | Patients with  pCR(primary lesions) | P | Patients with  non-pCR(lymph node) | Patients with  pCR(lymph node) | P |
| --- | --- | --- | --- | --- | --- | --- |
| Age(y) | 51.11±8.36 | 47.21±9.96 | 0.32* | 55±8.46 | 47.23±10.23 | 0.11* |
| BMI | 24.11±2.47 | 23.69±1.92 | 0.67* | 24.24±2.65 | 24.24±1.99 | 0.99* |
| Menstrual state |  |  | 1^†^ |  |  | 1^†^ |
| Postmenopause | 3(37.50%) | 5(62.50%) |  | 3(37.50%) | 5(62.50%) |  |
| Premenopause | 6(40.00%) | 9(60.00%) |  | 3(27.27%) | 8(72.73%) |  |
| Clinical T category |  |  | 0.82^#^ |  |  | 0.53^#^ |
| cT0 | - | - |  | 1(100.00%) | 0(0.00%) |  |
| cT1 | 1(50.00%) | 1(50.00%) |  | 1(50.00%) | 1(50.00%) |  |
| cT2 | 7(41.18%) | 10(58.82%) |  | 3(25.00%) | 9(75.00%) |  |
| cT3 | 1(25.00%) | 3(75.00%) |  | 1(25.00%) | 3(75.00%) |  |
| Clinical N category |  |  | 0.69^#^ |  |  | 0.66^#^ |
| cN0 | 2(40.00%) | 3(60.00%) |  | - | - |  |
| cN1 | 6(46.15%) | 7(53.85%) |  | 5(38.50%) | 8(61.50%) |  |
| cN2 | 0(0.00%) | 2(100.00%) |  | 0(0.00%) | 2(100.00%) |  |
| cN3 | 1(33.33%) | 2(66.67%) |  | 1(25.00%) | 3(75.00%) |  |
| AJCC Clinical stage |  |  | 1^†^ |  |  | 1^†^ |
| Stage II | 7(41.18%) | 10(58.82%) |  | 4(33.33%) | 8(66.67%) |  |
| Stage III | 2(33.33%) | 4(66.67%) |  | 2(28.57%) | 5(71.43%) |  |
| Ki-67 |  |  | 0.4^†^ |  |  | 0.18^†^ |
| High expression | 3(27.27%) | 8(72.73%) |  | 1(12.50%) | 7(87.50%) |  |
| Low expression | 6(50.00%) | 6(50.00%) |  | 5(45.45%) | 6(54.55%) |  |
| Histological grading |  |  | 1^†^ |  |  | 1^†^ |
| G2 | 3(42.86%) | 4(57.14%) |  | 2(33.33%) | 4(66.67%) |  |
| G3 | 6(37.50%) | 10(62.50%) |  | 4(30.77%) | 9(69.23%) |  |
| Molecular subtype |  |  | 0.71^#^ |  |  | 0.51^#^ |
| Luminal | 2(66.67%) | 1(33.33%) |  | 2(50.00%) | 2(50.00%) |  |
| HER2 positive | 4(33.33%) | 8(66.67%) |  | 2(18.18%) | 9(81.82%) |  |
| Triple-negative | 3(37.50%) | 5(62.50%) |  | 2(50.00%) | 2(50.00%) |  |
| Postoperative |  |  |  |  |  |  |
| NLR | 2.39(2.24~2.72) | 2.11(1.79~2.94) | 0.40^§^ | 2.72(2.5~4.71) | 2.17(1.74~2.57) | **0.03^§^** |
| PLR | 130.25(98.41~209.52) | 126.4(104.83~177.66) | 0.78^§^ | 202.57(121.61~307.13) | 122.22(102.62~131.13) | 0.27^§^ |
| LMR | 3.14(1.98~4.33) | 3.14(2.29~3.59) | 1^§^ | 1.98(1.58~3.75) | 3.26(2.56~3.63) | 0.24^§^ |
| SII | 399.14(301.86~621.73) | 399.14(263.63~626.17) | 0.93^§^ | 1117.51(506.96~1177.25) | 428.8(246.45~588.54) | 0.12^§^ |
| HALP score | 35.58(21.75~40.42) | 36.64(30.87~46.53) | 0.53^§^ | 23.91(15.98~33.2) | 40.41(33.77~47.15) | 0.12^§^ |
| PNI | 418.1±45.54 | 445.56±28.32 | 0.13* | 419.01(373.01~477.01) | 445.01(436.92~460.01) | 0.76^§^ |

Clinicopathologic Characteristics of Patients According to Primary Lesions and Lymph Nodes Pathologic Response

P values were calculated using Welch t test.

^#^P values were calculated using likelihood ratio test.

^†^P values were calculated using Fisher–Freeman–Halton exact test.

^§^P values were calculated using Mann–Whitney U test.

NLR = Neutrophil-to-Lymphocyte Ratio; PLR = Platelet-to-Lymphocyte Ratio; LMR = Lymphocyte-to-Monocyte Ratio; SII = Systemic Immune-Inflammation Index; PNI = Prognostic Nutritional Index.

Qualitative data are expressed as number (percentage); continuous data are expressed as mean ± SD or median (interquartile range: 25th–75th percentiles).

Online Resource 1 Table S2

Clinical Characteristics of Patients According to All Lesions Pathologic Response

| Characteristic | Patients with non-pCR | Patients with pCR | P |
| --- | --- | --- | --- |
| Age(y) | 52.2±8.6 | 47.21±9.96 | 0.20* |
| BMI | 24.34±2.44 | 23.69±1.92 | 0.50* |
| Menstrual state |  |  | 1^†^ |
| Postmenopause | 4(44.44%) | 5(55.56%) |  |
| Premenopause | 6(40.00%) | 9(60.00%) |  |
| Clinical T category |  |  | 0.71^#^ |
| cT0 | 1(100.00%) | 0(0.00%) |  |
| cT1 | 1(50.00%) | 1(50.00%) |  |
| cT2 | 7(41.18%) | 10(58.82%) |  |
| cT3 | 1(25.00%) | 3(75.00%) |  |
| Clinical N category |  |  | 0.70^#^ |
| cN0 | 2(40.00%) | 3(60.00%) |  |
| cN1 | 6(46.15%) | 7(53.85%) |  |
| cN2 | 0(0.00%) | 2(100.00%) |  |
| cN3 | 2(50.00%) | 2(50.00%) |  |
| AJCC Clinical stage |  |  | 1^†^ |
| Stage II | 7(41.18%) | 10(58.82%) |  |
| Stage III | 3(42.86%) | 4(57.14%) |  |
| Ki-67 |  |  | 0.24^†^ |
| High expression | 3(27.27%) | 8(72.73%) |  |
| Low expression | 7(53.85%) | 6(46.15%) |  |
| Histological grading |  |  | 1^†^ |
| G2 | 3(42.86%) | 4(57.14%) |  |
| G3 | 7(41.18%) | 10(58.82%) |  |
| Molecular subtype |  |  | 0.46^#^ |
| Luminal | 3(75.00%) | 1(25.00%) |  |
| HER2 positive | 4(33.33%) | 8(66.67%) |  |
| Triple-negative | 3(37.50%) | 5(62.50%) |  |
| Postoperative |  |  |  |
| NLR | 2.41(2.26~3.35) | 2.11(1.79~2.94) | 0.25^§^ |
| PLR | 154.2(106.29~206.05) | 126.4(104.83~177.66) | 0.58^§^ |
| LMR | 2.85(1.83~4.06) | 3.14(2.29~3.59) | 0.71^§^ |
| SII | 493.84(302.26~993.57) | 399.14(263.63~626.17) | 0.71^§^ |
| HALP score | 30.82(22.6~40.18) | 36.64(30.87~46.53) | 0.38^§^ |
| PNI | 424.39±47.32 | 445.56±28.32 | 0.23* |

*P values were calculated using Welch t test.

^#^P values were calculated using likelihood ratio test.

^†^P values were calculated using Fisher–Freeman–Halton exact test.

^§^P values were calculated using Mann–Whitney U test.

NLR = Neutrophil - Lymphocyte Ratio;PLR= Platelet - Lymphocyte Ratio;LMR = Lymphocyte - Monocyte Ratio;SII = Systemic Immune Inflammation Index;PNI = Prognostic Nutritional Index.

Qualitative data are number and percentage; continuous data are mean ± SD or median and 25th percentiles ~ 75th percentiles.

Online Resource 1 Table S3

PET Characteristics According to Pathologic Response in Primary Lesions and Axillary Lymph Nodes

| Imaging parameter | Subparameter | Patients with  non-pCR(primary lesions) | Patients with  pCR(primary lesions) | P | Patients with  non-pCR(lymph node) | Patients with  pCR(lymph node) | P |
| --- | --- | --- | --- | --- | --- | --- | --- |
| Pre-therapy |  |  |  |  |  |  |  |
| [^68^Ga]Ga-FAPI-04 | SUVmax | 17.18±6.64 | 15.26±6.7 | 0.49* | 9.79(8.13~12.25) | 7.92(4.56~10.08) | 0.08^§^ |
|  | SUVmean | 9.31±4.78 | 7.37±3.23 | 0.28* | 6.42(4.94~7.03) | 4.89(2.46~5.96) | 0.06^§^ |
|  | SULmax | 12.27±4.59 | 11.05±5.03 | 0.54* | 6.81(5.7~8.59) | 5.3(3.36~7.22) | 0.8^§^ |
|  | SULmean | 6.64±3.32 | 5.34±2.44 | 0.31* | 4.15±1.38 | 3.31±1.79 | 0.11* |
|  | SULpeak | 9.29±4.19 | 8.62±4.29 | 0.70* | 4.51(3.71~6.5) | 3.36(1.9~5.15) | 0.06^§^ |
|  | FTV(cm^3^) | 16.87±12.43 | 18.74±15.47 | 0.74* | 4.62±5.82 | 3.95±10.19 | 0.79* |
|  | TLF (SUV·cm^3^) | 93.34(60.19~174.32) | 112.99(47.51~244.94) | 1^§^ | 32.12±49.09 | 19.66±48.36 | 0.46* |
|  | TBR | 11.39±6.25 | 14.36±11.56 | 0.41* | 12.13±5.23 | 7.84±5.23 | **0.03*** |
| [^18^F]FDG | SUVmax | 8.27(6.44~10.26) | 12.73(6.76~15.76) | 0.40^§^ | 5.12(3.1~9.74) | 6.05(4.98~10.01) | 0.59^§^ |
|  | SUVmean | 3.63(3.42~4.11) | 5.21(3.67~7.85) | 0.26^§^ | 2.82(2.32~4.16) | 3.24(2.5~4.94) | 0.67^§^ |
|  | SULmax | 6.8±2.95 | 8.86±5.04 | 0.21* | 3.62(2.39~6.82) | 4.26(3.48~7.23) | 0.56^§^ |
|  | SULmean | 2.55(2.49~2.96) | 3.65(2.68~5.66) | 0.26^§^ | 1.98(1.64~2.92) | 2.37(1.76~3.61) | 0.62^§^ |
|  | SULpeak | 3.89(3.46~4.29) | 6.1(4~9.04) | 0.24^§^ | 2(1.64~3.59) | 2.29(1.67~4.04) | 0.97^§^ |
|  | MTV(cm^3^) | 14.02±10.53 | 12.8±10.3 | 0.78* | 1.65(0.47~6.2) | 1.23(0.76~1.7) | 0.95^§^ |
|  | TLG (SUV·cm^3^) | 45.04(25.92~78.38) | 50.66(24.34~117.83) | 0.85^§^ | 5.16(0.98~21.25) | 3.68(1.83~7.95) | 0.84^§^ |
|  | TBR | 5.9(4.18~7.86) | 6.94(4.33~14.15) | 0.46^§^ | 6.88(5.2~9.69) | 4.91(3.51~7.51) | 0.10^§^ |
| Post-therapy |  |  |  |  |  |  |  |
| [^68^Ga]Ga-FAPI-04 | SUVmax | 2.73(2.41~4.02) | 1.04(0.72~2.24) | <**0.01^§^** | 2.09(0.86~5.27) | 0.58(0.52~1.07) | <**0.01^§^** |
|  | SUVmean | 1.62(1.45~2.62) | 0.82(0.48~1.52) | **0.02^§^** | 1.6(0.53~3.16) | 0.41(0.36~0.44) | <**0.01^§^** |
|  | SULmax | 1.94(1.68~3.04) | 0.82(0.54~1.85) | **0.01^§^** | 1.54(0.6~3.69) | 0.42(0.37~0.78) | <**0.01^§^** |
|  | SULmean | 1.16(1.03~1.97) | 0.58(0.36~1.1) | <**0.01^§^** | 1.17(0.37~2.21) | 0.3(0.25~0.33) | <**0.01^§^** |
|  | SULpeak | 1.27(1.05~1.72) | 0.58(0.36~1.23) | **0.02^§^** | 0.92(0.37~1.84) | 0.3(0.25~0.33) | <**0.01^§^** |
|  | FTV(cm^3^) | 1.34(1.07~2.24) | 0(0~1.73) | 0.18^§^ | 0.62(0~0.8) | 0(0~0) | <**0.01^§^** |
|  | TLF (SUV·cm^3^) | 2.19(1.75~6.42) | 0(0~2.66) | 0.15^§^ | 0.89(0~2.51) | 0(0~0) | <**0.01^§^** |
|  | TBR | 2.64(2.32~3.83) | 1.8(1~2.53) | **0.01^§^** | 2.88(0.8~5.17) | 0.96(0.55~1.02) | **0.01^§^** |
| [^18^F]FDG | SUVmax | 1.58(1.53~1.95) | 1.58(1.25~1.77) | 0.27^§^ | 0.64(0.64~1.86) | 0.57(0.44~1.06) | 0.10^§^ |
|  | SUVmean | 1.17(1.13~1.48) | 1.12(0.7~1.32) | 0.29^§^ | 0.34(0.34~0.9) | 0.32(0.26~0.71) | 0.23^§^ |
|  | SULmax | 1.17(1.09~1.43) | 1.07(0.88~1.29) | 0.20^§^ | 0.45(0.45~1.4) | 0.42(0.34~0.71) | 0.16^§^ |
|  | SULmean | 0.88(0.82~1.07) | 0.85(0.5~0.94) | 0.21^§^ | 0.24(0.24~0.65) | 0.22(0.19~0.46) | 0.13^§^ |
|  | SULpeak | 0.86(0.84~1.06) | 0.82(0.52~0.96) | 0.23^§^ | 0.24(0.24~0.64) | 0.22(0.19~0.4) | 0.12^§^ |
|  | MTV(cm^3^) | 1.71(1.54~2.4) | 1.4(0.25~2.69) | 0.19^§^ | 0(0~0.45) | 0(0~0.35) | 0.89^§^ |
|  | TLG (SUV·cm^3^) | 2.45(1.83~3.99) | 1.75(0.3~2.74) | 0.16^§^ | 0(0~0.55) | 0(0~0.31) | 0.76^§^ |
|  | TBR | 2.98±1.49 | 2.43±1.21 | 0.34* | 1(0.96~2.99) | 0.84(0.68~1.41) | 0.11^§^ |
| MRI result |  |  |  | **0.02^†^** |  |  | **0.01^†^** |
| Negative |  | 2(15.38%) | 11(84.62%) |  | 2(11.76%) | 15(88.24%) |  |
| Positive |  | 8(66.67%) | 4(33.33%) |  | 6(60.00%) | 4(40.00%) |  |
| Pre- to post-therapy percentage change |  |  |  |  |  |  |  |
| [^68^Ga]Ga-FAPI-04 | ΔSUVmax% | 80.85(77.82~86.84) | 87.95(81.41~94.89) | 0.10^§^ | 79.55(47.31~88.32) | 88.69(79.52~94.12) | **0.02^§^** |
|  | ΔSUVmean% | 74.49(67.9~85.86) | 85.49(75.9~92.26) | 0.22^§^ | 78.14(52.4~81.13) | 89.75(81.72~93) | <**0.01^§^** |
|  | ΔSULmax% | 89.02(84.3~91.92) | 93.3(88.88~96.25) | 0.13^§^ | 79.14(47.37~88.15) | 88.72(79.7~94.16) | **0.03^§^** |
|  | ΔSULmean% | 75.69(65.49~85.93) | 85.48(76.46~92.47) | 0.20^§^ | 78.69(52.48~80.93) | 89.76(81.79~93.14) | <**0.01^§^** |
|  | ΔSULpeak% | 84.74(74.82~90.31) | 91.17(83.09~95.31) | 0.16^§^ | 82.17(62.25~86.63) | 89.49(83.38~93.78) | **0.03^§^** |
|  | ΔFTV% | 82.65(80.73~91.75) | 100(88.44~100) | **0.02^§^** | 85.64(65.43~100) | 100(100~100) | <**0.01^§^** |
|  | ΔTLF% | 97.28(91.01~98.8) | 100(96.69~100) | **0.03^§^** | 96.31(80.22~100) | 100(100~100) | <**0.01^§^** |
|  | ΔTBR% | 66.78(60.44~79.44) | 76.42(62.76~93.6) | 0.31^§^ | 79.57(66.13~85.77) | 87.7(79.79~92.12) | 0.13^§^ |
| [^18^F]FDG | ΔSUVmax% | 77.5(73.74~84.82) | 87.72(83.43~89.84) | 0.06^§^ | 79.25(76.59~88.86) | 90.48(81.67~92.86) | 0.09^§^ |
|  | ΔSUVmean% | 64.97±19.54 | 78.15±14.18 | 0.09* | 85.13(75.92~89.47) | 87.83(83.6~92.52) | 0.29^§^ |
|  | ΔSULmax% | 77.85(72.46~84.37) | 87.97(83.62~90.08) | **0.04^§^** | 80.43(76.26~88.44) | 90.3(82.1~92.91) | 0.09^§^ |
|  | ΔSULmean% | 64.8±18.96 | 78.23±14.12 | 0.07* | 84.98(77.25~89.4) | 88.12(83.7~92.54) | 0.29^§^ |
|  | ΔSULpeak% | 77.59(74~80.13) | 88.19(81.77~90.15) | <**0.01^§^** | 84.59(77.96~92.34) | 88.65(84.66~92.97) | 0.27^§^ |
|  | ΔMTV% | 81.52(74.37~93.39) | 86.38(66.19~99.18) | 0.76^§^ | 100(95.22~100) | 100(88.25~100) | 0.99^§^ |
|  | ΔTLG% | 95.42(90.59~97.72) | 97.28(93.6~99.78) | 0.28^§^ | 100(98.5~100) | 100(98.87~100) | 0.94^§^ |
|  | ΔTBR% | 52.36(37.27~69.13) | 74.41(69.68~79.14) | **0.01^§^** | 79.51(64.4~87.16) | 82.05(74.42~87.36) | 0.97^§^ |

*P values were calculated using Welch t test.

^†^P values were calculated using Fisher–Freeman–Halton exact test.

^§^P values were calculated using Mann–Whitney U test.

Qualitative data are presented as number and percentage; continuous data are expressed as mean ± SD or median and 25th-75th percentiles. d indicates percentage change from pre-therapy to post-therapy.

Online Resource 1 Table S4

Summary of Benjamini-Hochberg False Discovery Rate Correction for Exploratory Univariable Analyses

| Family of comparisons | Nominally significant finding(s) before FDR | FDR-adjusted q value | Status after FDR |
| --- | --- | --- | --- |
| Primary-lesion imaging comparisons | Post-therapy FAPI SUVmean and SULpeak; FAPI ΔFTV% and ΔTLF%; FDG ΔSULmax% | 0.163 | Not retained |
| Primary-lesion imaging comparisons | Post-therapy FAPI SUVmax, SULmax, SULmean, and TBR; FDG ΔSULpeak%, and ΔTBR% | 0.041 | Retained |
| Axillary nodal imaging comparisons | Post-therapy FAPI SUVmax, SUVmean, SULmax, SULmean, SULpeak, FTV, TLF, and TBR; nodal MRI result; FAPI ΔSUVmean%, ΔSULmean%, ΔFTV%, and ΔTLF% | 0.038 | Retained |
| Axillary nodal imaging comparisons | Baseline FAPI TBR; FAPI ΔSUVmax%, ΔSULmax%, and ΔSULpeak% | 0.070–0.087 | Not retained |
| Overall-lesion imaging comparisons | Post-therapy FAPI TLR | 0.669 | Not retained |
| HER2-positive subgroup | Pre-therapy FAPI SUVmean and SULmean; post-therapy FDG MTV; FDG ΔTBR% | 0.040 | Retained within subgroup family |
| HER2-negative subgroup | FAPI ΔSUVmax% and ΔSULmax%; post-therapy FAPI SUVmax, SUVmean, and SULmean | 0.049 | Retained within subgroup family |

Benjamini-Hochberg false discovery rate correction was applied separately to predefined families of univariable comparisons. P values reported as less than 0.01 in the source tables were conservatively entered as 0.01 for q-value estimation.

Online Resource 1 Table S5

PET Characteristics of Patients According to Pathologic Response

| Imaging parameter | Subparameter | Patients with  non-pCR | Patients with  pCR | P |
| --- | --- | --- | --- | --- |
| Pre-therapy |  |  |  |  |
| [^68^Ga]Ga-FAPI-04 | SUVmax (g/mL) | 16.66±6.44 | 16.85±6.23 | 0.94* |
|  | SUVpeak (g/mL) | 12.88±5.65 | 13.4±5.2 | 0.82* |
|  | SULmax (g/mL) | 9.77(8.99~15.4) | 12.5(9.64~16.24) | 0.82^§^ |
|  | SULpeak (g/mL) | 8.96±4.11 | 9.52±3.95 | 0.73* |
|  | FTV(cm3) | 19.57(12.68~32.74) | 23.13(10.15~33.23) | 0.91^§^ |
|  | TLF(g/mL3cm3) | 122.83(72.94~342.2) | 161.46(74.53~295.62) | 0.91^§^ |
|  | TLR | 16.39±6.35 | 18.29±9.66 | 0.57* |
| [^18^F]FDG | SUVmax (g/mL) | 10.58±4.27 | 13.39±7.34 | 0.26* |
|  | SUVpeak (g/mL) | 5.6(5~9.27) | 9.83(6.54~13.34) | 0.42^§^ |
|  | SULmax (g/mL) | 7.6±3.03 | 9.63±5.21 | 0.25* |
|  | SULpeak (g/mL) | 4.27(3.49~6.4) | 6.39(4.87~9.52) | 0.38^§^ |
|  | MTV(cm3) | 16.24(8.89~31.95) | 13.7(9.68~25.41) | 0.86^§^ |
|  | TLG(g/mL3cm3) | 55.15(31.46~196.05) | 110.32(39.69~152.22) | 0.73^§^ |
|  | TLR | 4.06±1.94 | 4.84±2.8 | 0.43* |
| Post-therapy |  |  |  |  |
| [^68^Ga]Ga-FAPI-04 | SUVmax (g/mL) | 2.61(2.26~6.37) | 2.11(1.04~2.52) | 0.08^§^ |
|  | SUVpeak (g/mL) | 1.68(1.42~3.52) | 1.37(0.7~1.62) | 0.09^§^ |
|  | SULmax (g/mL) | 1.86(1.69~4.7) | 1.37(0.73~1.85) | 0.08^§^ |
|  | SULpeak (g/mL) | 1.2(1.01~2.52) | 1.02(0.49~1.22) | 0.12^§^ |
|  | FTV(cm3) | 1.65(1.11~3.12) | 1.72(0~2.19) | 0.56^§^ |
|  | TLF(g/mL3cm3) | 2.63(2.06~7.58) | 2.58(0~3.16) | 0.28^§^ |
|  | TLR | 2.97±2.3 | 1.36±0.69 | **0.04*** |
| [^18^F]FDG | SUVmax (g/mL) | 1.69(1.62~2.26) | 1.65(1.43~2.09) | 0.43^§^ |
|  | SUVpeak (g/mL) | 1.23(1.17~1.46) | 1.19(0.87~1.37) | 0.22^§^ |
|  | SULmax (g/mL) | 1.21(1.18~1.63) | 1.17(1.04~1.51) | 0.37^§^ |
|  | SULpeak (g/mL) | 0.91(0.85~1.04) | 0.88(0.6~0.97) | 0.22^§^ |
|  | MTV(cm3) | 2.36(1.71~3.15) | 2.3(1.16~3.52) | 0.56^§^ |
|  | TLG(g/mL3cm3) | 2.96(2.62~4.5) | 2.96(1.18~5) | 0.54^§^ |
|  | TLR | 0.59(0.57~0.68) | 0.57(0.52~0.69) | 0.37^§^ |
| Pre- to post-therapy percentage change |  |  |  |  |
| [^68^Ga]Ga-FAPI-04 | △SUVmax% | 82.47(56.46~89.49) | 86.86(82.33~90.27) | 0.30^§^ |
|  | △SUVpeak% | 86.68(70.03~91.52) | 89.25(83.78~94.74) | 0.33^§^ |
|  | △SULmax% | 81.94(54.03~89.39) | 86.71(82.44~90.78) | 0.25^§^ |
|  | △SULpeak% | 86.96(68.4~91.42) | 87.44(84.26~94.98) | 0.39^§^ |
|  | △FTV% | 87.36±11.97 | 92.65±7.14 | 0.22* |
|  | △TLF% | 98.5(92.59~99.28) | 98.61(96.57~100) | 0.23^§^ |
|  | △TLR% | 91.11(67.56~92.57) | 90.87(89.02~94.98) | 0.46^§^ |
| [^18^F]FDG | △SUVmax% | 76.27(72.66~87.11) | 85.76(77.87~89.09) | 0.33^§^ |
|  | △SUVpeak% | 77.15(71.79~86.38) | 87.95(81.89~92.15) | 0.17^§^ |
|  | △SULmax% | 76.69(72.41~86.92) | 86.12(77.86~89.14) | 0.33^§^ |
|  | △SULpeak% | 76.12±13.42 | 84.55±10.95 | 0.11* |
|  | △MTV% | 84.8(76.07~92.36) | 90.32(74.3~96.84) | 0.86^§^ |
|  | △TLG% | 96.63(89.65~98.06) | 96.1(95.07~99.38) | 0.32^§^ |
|  | △TLR% | 78.42(73.75~87.96) | 85.96(79.08~88.58) | 0.57^§^ |

*P values were calculated using Welch t test.

^§^P values were calculated using Mann–Whitney U test.

Continuous data are mean ± SD or median and 25th percentiles ~ 75th percentiles.

Online Resource 1 Table S6

Subgroup Analysis of PET Characteristics According to Pathologic Response in Primary Lesions (Stratified by HER2 Expression)

| Imaging parameter | Subparameter | Patients with  non-pCR(HER2+) | Patients with  pCR(HER2+) | P | Patients with  non-pCR(HER2-) | Patients with  pCR(HER2-) | P |
| --- | --- | --- | --- | --- | --- | --- | --- |
| [^68^Ga]Ga-FAPI-04 | Pre-therapy SUVmean | 13.83±3.74 | 7.83±3.83 | **0.04*** | / | / | / |
|  | Pre-therapy SULmean | 9.78±2.58 | 5.66±2.88 | **0.04*** | / | / | / |
|  | ΔSUVmax% | / | / | / | 77.85(45.8~80.14) | 90.71(88.53~91.91) | **<0.01^§^** |
|  | ΔSULmax% | / | / | / | 79.74±11.52 | 92.24±5.21 | **0.046*** |
|  | Post-therapy SUVmax | / | / | / | 4.76±3.23 | 1.37±0.85 | **0.049*** |
|  | Post-therapy SUVmean | / | / | / | 2.63±1.38 | 1.01±0.66 | **0.04*** |
|  | Post-therapy SULmean | / | / | / | 1.94±1.04 | 0.74±0.49 | **0.04*** |
| [^18^F]FDG | Post-therapy MTV(cm^3^) | 1.8(1.54~2.18) | 0.51(0~1.4) | **0.03*** | / | / | / |
|  | ΔTBR% | 42.55(34.92~52.08) | 74.41(65.17~76.46) | **0.03*** |  |  |  |

*P values were calculated using Welch t test.

^§^P values were calculated using Mann–Whitney U test.

Online Resource 1 Table S7

Subgroup Analysis of Diagnostic Performance of Imaging Parameters for Prediction of pCR in Primary Lesions (Stratified by HER2 Expression)

| HER2 Expression | Parameter | Subparameter | Cutoff | AUC | P | 95%CI | Sensitivity(%) | Specificity(%) | Accuracy(%) |
| --- | --- | --- | --- | --- | --- | --- | --- | --- | --- |
| HER2 Positive | [^68^Ga]Ga-FAPI-04 | Pre-therapy SUVmean | 13.295 | 0.861 | **0.04** | 0.602-1.000 | 75.00% | 100.00% | 92.31% |
|  | [^18^F]FDG | Post-therapy  MTV(cm^3^) | 1.49 | 0.889 | **0.03** | 0.684-1.000 | 100.00% | 88.90% | 92.31% |
| HER2 Negative | [^68^Ga]Ga-FAPI-04 | ΔSUVmax% | 87.46 | 0.944 | **<0.01** | 0.814-1.000 | 83.30% | 100.00% | 91.67% |
|  | [^18^F]FDG | / | / | / | **/** | / | / | / | / |

AUC = Area Under the Curve.
