# Supplementary material for: Prospective comparison of [68Ga]Ga-FAPI-04 PET/MRI, [18F]FDG PET/CT, and contrast-enhanced MRI for predicting pathologic complete response after neoadjuvant chemotherapy in breast cancer
Source: Eur J Nucl Med Mol Imaging. 2026 Jul 23;53(12):7221–32. doi: 10.1007/s00259-026-08091-y (PMC13633318; doi:10.1007/s00259-026-08091-y)
Supplement: Supplementary file 2 [file 259_2026_8091_MOESM2_ESM.docx]

Online Resource 2 Fig. S1. Least absolute shrinkage and selection operator (LASSO) coefficient profiles for primary-lesion analyses. A and B show the [^68^Ga]Ga-FAPI-04 coefficient profile and cross-validation curve; C and D show the corresponding [^18^F]FDG plots.

Online Resource 2 Fig. S2. Least absolute shrinkage and selection operator (LASSO) coefficient profiles for axillary nodal [^68^Ga]Ga-FAPI-04 analyses. A shows the coefficient profile and B shows the cross-validation curve.

Online Resource 2 Fig. S3. Receiver-operating-characteristic (ROC) curves of optimal imaging parameters for predicting pathologic complete response (pCR). A, primary-lesion curves for post-therapy [^68^Ga]Ga-FAPI-04 SUVmax, [^18^F]FDG ΔTBR%, and contrast-enhanced MRI. B, nodal curves for post-therapy [^68^Ga]Ga-FAPI-04 SULmean, [^18^F]FDG ΔSULmax%, and contrast-enhanced MRI. FDG = fluorodeoxyglucose; MRI = magnetic resonance imaging; SULmax = maximum standardized uptake value normalized by lean body mass; SULmean = mean standardized uptake value normalized by lean body mass; SUVmax = maximum standardized uptake value; TBR = tumor-to-background ratio.

Online Resource 2 Fig. S4. Receiver-operating-characteristic (ROC) curves of optimal imaging parameters for pathologic complete response (pCR) prediction in HER2-defined subgroups. A, HER2-positive subgroup. B, HER2-negative subgroup. HER2 = human epidermal growth factor receptor 2.
